# Assessment the awareness of vitamin D deficiency among the general population in Syria: an online cross-sectional study

**DOI:** 10.1186/s12889-024-18376-2

**Published:** 2024-04-01

**Authors:** Hidar Alibrahim, Sarya Swed, Haidara Bohsas, Yasmeen Abouainain, Nagham Jawish, Rehab Diab, Angela Ishak, Heba Haj Saleh, Mohamad Nour Nasif, Rahaf Arafah, Wajih Abboud Abboud, Asma’a Horan Suliman, Bisher Sawaf, Wael Hafez

**Affiliations:** 1 Faculty of Medicine, Aleppo University, Aleppo, Syria; 2 Faculty of Medicine, University of Jordan, Amman, Jordan; 3 Faculty of Medicine, Damascus University, Damascus, Syria; 4 Faculty of Medicine, Al-Azhar University, Cairo, Egypt; 5 European University School - School of Medicine, Nicosia, Cyprus; 6 Department of Internal Medicine, Hamad Medical Corporation, Doha, Qatar; 7 NMC Royal Hospital, 16th Street, Khalifa City, Abu Dhabi UAE; 8 Medical Research Division, Department of Internal Medicine, The National Research Centre, Cairo, Egypt

**Keywords:** Vitamin D deficiency, Syria, Awareness, Cross-sectional Study

## Abstract

**Background:**

Vitamin D deficiency is an importance preventable problem in the global and associates with lack levels of awareness about vitamin D. According to prior studies, in the Arab world, there is low of knowledge and awareness toward vitamin D deficiency. The target of our study is evaluating the knowledge level about vitamin D deficiency and determining the associated factors with levels of awareness of its.

**Method:**

This online cross-sectional study was performed in Syria between 25 February to 29 March 2023 to assess the levels of knowledge about vitamin D deficiency among general Syrian population. The study’s survey was obtained from previously published research and we conducted a pilot study to assure the validity and clarity questionnaire. All Syrian individuals aged 18 or older who were able to read and write and willing to participate were included, while, non-Syrian nationality individuals and all medical staff (doctors, nurses, and medical students…), as well, those under 18 age were excluded. The questionnaire consisted of 23 questions separated into four categories. The first section was sociodemographic information of the study population. The second section measured the level awareness of the study population regarding the benefits of vitamin D. In addition, the third and fourth part evaluated knowing of the respondents about sources of and toxicity consequences of vitamin D. The data were analyzed by utilizing multivariate logistic regression in IBM, SPSS V.28 version.

**Results:**

Overall, 3172 of the study population accepted to participate in this research and 57.9% the majority of them were aged in the range among 18 and 28. While, the average age of the respondents were 30.80 ± 11.957. Regarding with the awareness toward knowledge of advantages and source of vitamin D and outcomes of vitamin D toxicity. Most of the participants mentioned that vitamin D is used to treat bone disease and rickets and contributes in maintaining calcium and phosphates (91.4% and 84.6%, respectively). Whereas, more than half of them reported that sun exposure does not cause vitamin D poisoning and that vegetarians are more likelihood to have vitamin D than non-vegetarians, (54.1% and 54.9%, respectively). Only, age and occupation out of nine predictors variables were significantly correlated with adequate knowledge of Vitamin D (p-value < 0.05). The respondents aged more than 60 years were high probability to have good recognition of Vitamin D than participants aged between 18 and 28 years. (OR = 7.95). Retired participants have shown lower aware of Vitamin D 0.38 times than students.

**Conclusion:**

Our research revealed that most of the participated individuals have sufficient comprehension about vitamin D, despite, there were significant gap. Health education via programs by government health-care agencies, NGOs and social workers is necessary to increase the awareness and knowledge toward benefits, source, deficiency and toxicity of vitamin D to avoid injury several diseases such as rickets.

**Supplementary Information:**

The online version contains supplementary material available at 10.1186/s12889-024-18376-2.

## Introduction

Vitamin D deficiency or insufficiency affects one billion individuals globally, constituting a substantial and preventable public health concern [1]. The two primary forms of vitamin D, D2 and D3, play vital roles in human health. Vitamin D3, or cholecalciferol, is primarily synthesized by skin cells in response to ultraviolet-B light (UVB) and is present in animal-derived food, while vitamin D2, or ergocalciferol, is sourced from plants such as mushrooms and yeast [2]. Evidence suggested that skin-derived vitamin D possesses a longer half-life in the bloodstream compared to diet-derived vitamin D [3]. Factors such as sunscreen use, aging, and darker skin can potentially limit the synthesis of vitamin D in the skin [4–6]. While natural sources of vitamin D include the yolk of eggs, fatty fish like salmon, and organ meats such as liver, fortified products like milk and cereals are prevalent in several countries [7].

Beyond its pivotal role in calcium homeostasis and bone development, vitamin D deficiency in children may lead to rickets, while in adults, it can result in osteomalacia [8]. Emerging evidence indicates the potential benefits of vitamin D in immunological function, cardiovascular health, cancer prevention, and skeletal health [9].

Vitamin D deficiency is defined by a serum 25(OH) D level below 20 ng/mL, while insufficiency ranges from 21 to 29 ng/mL without apparent clinical symptoms [10]. This insufficiency has been associated with various acute and chronic conditions, including preeclampsia, childhood dental caries, gingivitis, autoimmune and infectious diseases, cardiovascular ailments, fatal malignancies, type 2 diabetes, and neurological disorders [11].

Certain medical conditions, such as malabsorption, short bowel syndrome, and individuals with cancer, elevate the risk of vitamin D deficiency [12]. Those using anticonvulsants, rifampicin, or highly active antiretroviral medications are also at a heightened risk [12]. Additionally, individuals who abstain from meat consumption and those with photosensitive skin diseases face increased vulnerability [12]. Addressing low vitamin D status, the World Health Organization-Food and Agriculture Organization (WHO-FAO) recommends a combination of diversified food intake, fortification, and supplementation [13].

A recent study revealed a correlation between vitamin D deficiency, low awareness about vitamin D, and insufficient intake of vitamin supplements [14]. Similarly, previous research in the Arab world has identified a lack of general population knowledge and awareness regarding vitamin D deficiency, highlighting the need for educational programs to enhance awareness [15, 16]. Several studies emphasize the critical role of acquiring appropriate knowledge in mitigating vitamin D deficiency and promoting overall health [17].

Crucially, the need to enhance public understanding of the importance of vitamin D and the adverse effects of insufficient vitamin D at all life stages cannot be overstated [18]. In Syria, a substantial 58.2% of adults are estimated to possess a serum 25(OH) D level below 25 nmol/L, underscoring the pervasive and critical nature of vitamin D deficiency within the population [19]. Recognizing the urgency of this widespread issue, the assessment of the general public’s awareness and knowledge of vitamin D deficiency in Syria becomes paramount. This study stands as a pioneering effort, breaking new ground as the first systematic evaluation of the knowledge and awareness of vitamin D deficiency among the Syrian population.

Our primary objectives encompass a comprehensive determination of knowledge levels regarding vitamin D deficiency within this population. We strive to delve into the intricacies of vitamin D awareness and knowledge, aiming to identify potential associations with various demographic and socioeconomic factors. By shedding light on these objectives, our study seeks to provide nuanced insights that extend beyond mere prevalence statistics. The aim is to inform targeted interventions and contribute to the development of tailored and effective public health strategies aimed at mitigating the prevalence of vitamin D deficiency in Syria.

In essence, this research aspires to play a pivotal role in not only quantifying the extent of the issue but also unraveling the underlying factors that contribute to vitamin D deficiency in the Syrian context. Through these comprehensive endeavors, we are optimistic about making substantial strides in reducing the prevalence of vitamin D deficiency and improving overall public health outcomes in Syria.

## Methods

### Study design and setting

To assess the knowledge of vitamin D deficiency and evaluate awareness regarding its sources and toxicity among the Syrian population, we conducted a web-based cross-sectional study from February 25 to March 29, 2023. Prior to participation, participants were presented with detailed information about the study’s objective on the initial page of the Google Form survey, ensuring transparency and obtaining informed consent. Participants were informed of their right to withdraw, and we guaranteed the protection of their personal information, with the assurance that only completed surveys would undergo statistical analysis.

The study included Syrian individuals aged 18 or older who could read and write and were willing to participate. Excluded from the study were individuals with non-Syrian nationality, those under 18, and participants in the medical field, such as doctors, nurses, and medical students. The exclusion of healthcare professionals aimed to focus on the general public’s knowledge and awareness of vitamin D deficiency, minimizing potential bias introduced by specialized knowledge. The survey was distributed exclusively to individuals meeting the predefined inclusion criteria, such as being part of the general Syrian population. We utilized targeted online platforms and channels to reach our intended demographic while minimizing the risk of inclusion of individuals from excluded groups. Additionally, the survey introduction explicitly outlined the eligibility criteria, and participants were informed that only those meeting these criteria should proceed.

The study items were designed based on a comprehensive and validated scale used in prior research conducted in Jeddah, Saudi Arabia [20]. Subsequently, the questionnaire was translated into Arabic for respondents’ convenience. Sampling was conducted using convenience and snowball tactics. The questionnaire, created on the Google Form website, was distributed across social media platforms, including Facebook, WhatsApp, and Telegram, with the assistance of specific collaborators from various medical Syrian colleges (Data Collection Group). The data collection team, supervised by an appointed supervisor with access to the Google Form website, ensured the systematic implementation of the study protocol.

### Sample size and sampling method

The sample size was determined using Calculator.net, accessible at [21]. Considering the estimated Syrian population of approximately 20,098,251 in 2019 [22], we conducted a statistical power analysis for sample size calculation. With a population proportion assumption of 50%, a margin of error set at 0.05, and a confidence level of 95%, the recommended sample size was 385. Remarkably, the final sample size on the Google Form website exceeded this estimate, reaching 2625 participants.

### Measures

The study questionnaire comprised four distinct sections, encompassing a total of 23 questions. The first part gathered respondents’ characteristics, while the second section focused on assessing awareness regarding the benefits of vitamin D. The third and fourth segments evaluated awareness regarding sources of vitamin D and knowledge of consequences related to vitamin D toxicity, respectively.

For each knowledge item, respondents provided either a “yes” or “no” response. The total knowledge score ranged from 0 to 14, with 1 point awarded for each correct response and 0 points for each incorrect one.

#### Socioeconomic characteristics

This segment comprised 9 questions aimed at capturing key demographic information from each respondent. Variables such as age, gender, educational level, economic status, marital status, and the number of children were essential components in understanding the socioeconomic characteristics of the participants.

#### Knowledge of vitamin D benefits

Five questions in this section were designed to gauge participants’ understanding of the benefits of vitamin D. Participants were queried on whether vitamin D is used in treating bone diseases and rickets, its role in maintaining calcium and phosphates, and its significance in bone and teeth maintenance.

#### Knowledge of vitamin D sources

This section included eight items aimed at assessing respondents’ knowledge of vitamin D sources. Questions covered topics such as the role of sun exposure in vitamin D production in the skin, whether the use of sunscreen creams could lead to vitamin D deficiency, and whether a fat-free diet might contribute to vitamin D deficiency.

#### Knowledge of vitamin D toxicity consequences

Participants were asked a single question in this section, specifically inquiring whether hypercalcemia was associated with vitamin D toxicity.

### Pilot study

To assess the validity and clarity of the questionnaire, a preliminary version was administered to 50 randomly selected members of the public. The survey items were refined based on insights gained from this pretest. Following modifications, a pilot test involving another set of 50 participants was conducted, ensuring high levels of internal consistency (Cronbach’s alpha ranged from 0.712 to 0.861). After confirming the reliability and appropriateness of the questionnaire through the pilot study, it was disseminated for the main data collection.

### Declarations

#### Informed consent and ethical statement

The online survey, hosted on Google Forms, was made accessible to all respondents via a provided URL. Informed consent was obtained electronically, with participants explicitly confirming their willingness to partake in the study before proceeding with the questionnaire. A comprehensive explanation of the research objectives was presented on the initial page. Each response was securely stored in an online database, and completion of the questionnaire typically required five to twelve minutes.

Ethical approval for the study was obtained from the Syrian Ethical Society for Scientific Research (IRB: TJ/O-39), and ethical clearance was granted by Aleppo University. All procedures adhered to relevant guidelines and regulations, including the Declaration of Helsinki.

### Statistical analysis

Following the data collection through Google Forms, data extraction to an Excel sheet was conducted, and subsequent analysis was performed using IBM SPSS V.28. Sociodemographic information was presented for the study population using statistical descriptive measures, frequencies, and percentages for categorical data. Mean and standard deviation were employed for continuous data, and the Kruskal-Wallis test was utilized to identify factors influencing knowledge regarding Vitamin D (including benefits, sources, toxicity, and overall awareness). Statistical significance was determined at a p-value of ≤ 0.05.

To assess factors influencing awareness of Vitamin D, a multivariate logistic regression test was conducted. Individuals scoring above 50% were considered to have an adequate level of awareness. The determination of awareness levels adhered to established references in the field [23, 24], ensuring a robust and validated approach to categorization.

## Results

### Participant characteristics

In this study, a total of 3172 participants engaged with our survey, resulting in 2625 completed surveys (response rate: 82.8%), while 547 respondents chose not to participate. Participant demographics revealed significant variations across multiple subgroups.

- **Age**: The mean age of study participants was 30.80 ± 11.957 years. More than half of the participants (1521, 57.9%) fell within the age range of 18 to 28 years (Table 1).
- **Gender**: A substantial majority of respondents were females, constituting 74.5% (1955) of the study population (Table 1**).**
- **Residence**: Regarding residential location, 58.2% (1096) of participants lived in urban areas (Table 1).
- **Marital Status**: Marital status distribution showed that 40.8% (1071) of respondents were married (Table 1).
- **Education**: Educational attainment varied, with almost two-thirds of participants (1707, 65.0%) holding a university degree (Table 1).
- **Occupation**: In terms of occupation, 16.3% (429) of participants reported working in the public sector (Table 1).

**Table 1:** Socioeconomic demographic information of the participants that completed the survey

Variable	Categories	N	%
Age	Mean ± SD	30.80 ± 11.957	
Age groups	18–28	1521	57.9%
	29–39	517	19.7%
	40–50	380	14.5%
	51–60	144	5.5%
	More than 60	63	2.4%
Gender	Female	1955	74.5%
	Male	670	25.5%
Living location	Countryside	1096	41.8%
	City	1529	58.2%
Marital status	Single	1443	55.0%
	Married	1071	40.8%
	Widowed	53	2.0%
	Divorced	58	2.2%
Level of education	Illiterate	14	0.5%
	Primary school	43	1.6%
	Middle school	117	4.5%
	secondary school	583	22.2%
	University	1707	65.0%
	PhD	161	6.1%
Monthly income	High	47	1.8%
	Good	879	33.5%
	Middle	1514	57.7%
	Low	185	7.0%
Occupation	Student	843	32.1%
	Public sector	429	16.3%
	Private Job	716	27.3%
	Retired	110	4.2%
	Non-working	362	13.8%
	Other	164	6.3%
Chronic diseases	No	2349	89.5%
	Yes	276	10.5%
Have children	No	1637	62.4%
	Yes	988	37.6%

### Participants’ awareness toward knowledge of advantages and source of vitamin D and outcomes of vitamin D toxicity

The majority of participants (91.4% and 84.6%) correctly acknowledged that vitamin D is utilized to treat bone disease and rickets and is vital in maintaining calcium and phosphates. However, 32.8% of respondents did not recognize the role of Vitamin D in strengthening muscles, and 62.2% did not correctly identify that it is predominantly found in animal meat rather than vegetables and fruits. Additionally, more than half of the respondents (54.1% and 54.9%) believed that frequent sun exposure does not lead to vitamin D poisoning and that vegetarians are more likely to experience vitamin D deficiency than non-vegetarians, respectively (Table 2).

**Table 2:** Participants’ awareness toward knowledge of advantages and source of vitamin D and outcomes of vitamin D toxicity

Variable	Categories	N	%
Vitamin D is used to treat bone disease and rickets	No	225	8.6%
	Yes	2400	91.4%
Vitamin D is important in the maintenance of calcium and phosphates	No	405	15.4%
	Yes	2220	84.6%
Vitamin D is important in the maintenance of bone and teeth	No	311	11.8%
	Yes	2314	88.2%
Vitamin D helps to strengthen immunity	No	528	20.1%
	Yes	2097	79.9%
Vitamin D helps to strengthen muscles	No	861	32.8%
	Yes	1764	67.2%
Sun exposure encourages vitamin D production in the skin	No	115	4.4%
	Yes	2510	95.6%
Vitamin D is found in animal meat but not in vegetables and fruits	No	1633	62.2%
	Yes	992	37.8%
People residing in cloudy areas are more prone to vitamin D deficiency	No	358	13.6%
	Yes	2267	86.4%
Frequent sun exposure does not lead to vitamin D poisoning	No	1205	45.9%
	Yes	1420	54.1%
Use of sunscreen creams may be a cause of vitamin D deficiency	No	1614	61.5%
	Yes	1011	38.5%
A fat-free diet may be a cause of vitamin D deficiency	No	1240	47.2%
	Yes	1385	52.8%
Dark skin is more prone to vitamin D deficiency than fairer skin	No	1854	70.6%
	Yes	771	29.4%
Vegetarians are more likely to have vitamin D deficiency than nonvegetarians	No	1183	45.1%
	Yes	1442	54.9%
Consequences of vitamin D toxicity in Hypercalcemia	No	990	37.7%
	Yes	1635	62.3%

### The mean +-SD for benefits, source, toxicity and overall scores of the characteristic’s participants toward vitamin D

The benefits score demonstrated statistically significant correlations with six baseline characteristics (p-value < 0.05). Participants aged > 60 exhibited higher benefit scores compared to other age groups (mean = 4.365). Females identified more benefits of vitamin D than males, and respondents with Ph.D. degrees had a superior average benefit score compared to other education levels.

For the source’s score, six baseline characteristics were found to be associated (p-value < 0.05). Males had a higher awareness score toward vitamin D sources than females (mean = 4.839). Respondents with chronic diseases demonstrated greater awareness of vitamin D sources (mean = 4.913), while those with children had a higher level of awareness about vitamin D sources compared to those without (mean = 4.644) (Table 3).

Significant relationships were observed between five baseline characteristics and the overall knowledge score (p-value < 0.05). Widowed participants exhibited higher overall knowledge of vitamin D than other marital statuses (mean = 10.283). Surprisingly, respondents with stated low monthly income had a higher average overall knowledge score toward vitamin D (mean = 9.735) (Table 3).

**Table 3:** The mean +-SD for benefits, source, toxicity and overall scores of the characteristics participants toward vitamin D

Variable	Categories	Benefits		Sources		Toxicity		Overall	
		Mean	Standard deviation	Mean	Standard deviation	Mean	Standard deviation	Mean	Standard deviation
Age groups	18–28	3.978	0.990 *	4.312	1.710 *	0.615	0.487	8.904	2.313 *
	29–39	4.319	0.917	4.544	1.727	0.634	0.482	9.497	2.296
	40–50	4.308	0.971	4.874	1.804	0.658	0.475	9.839	2.346
	51–60	4.167	1.010	5.014	1.701	0.604	0.491	9.785	2.413
	More than 60	4.365	0.829	5.032	1.481	0.556	0.501	9.952	1.987
Gender	Female	4.141	0.947 *	4.376	1.690 *	0.617	0.486	9.134	2.264 *
	Male	4.030	1.079	4.839	1.830	0.640	0.480	9.509	2.547
living location	Countryside	4.150	0.975	4.521	1.761	0.624	0.485	9.295	2.319
	City	4.086	0.989	4.475	1.722	0.622	0.485	9.183	2.363
marital status	Single	3.997	0.986 *	4.389	1.734 *	0.611	0.488	8.997	2.353 *
	Married	4.247	0.962	4.604	1.735	0.643	0.479	9.495	2.303
	Widowed	4.396	0.906	5.264	1.700	0.623	0.489	10.283	2.324
	Divorced	4.241	1.014	4.397	1.685	0.534	0.503	9.172	2.202
Level of education	Illiterate	3.500	1.829 *	5.000	1.569	0.357	0.497	8.857	2.248
	Primary school	3.953	1.045	5.349	1.378	0.581	0.499	9.884	2.163
	Middle school	4.068	1.244	4.932	1.955	0.590	0.494	9.590	2.691
	secondary school	4.051	0.997	4.581	1.692	0.636	0.481	9.269	2.310
	University	4.118	0.957	4.378	1.732	0.628	0.483	9.124	2.335
	PhD	4.404	0.817	4.820	1.746	0.578	0.495	9.801	2.249
Monthly income	High	4.085	1.080	4.574	1.778 *	0.638	0.486	9.298	2.245 *
	Good	4.102	0.976	4.342	1.705	0.619	0.486	9.064	2.300
	Middle	4.119	0.964	4.520	1.724	0.624	0.485	9.262	2.327
	Low	4.114	1.144	4.989	1.908	0.632	0.483	9.735	2.642
Occupation	Student	3.986	1.002	4.399	1.718	0.636	0.481	9.020	2.334
	Public sector	4.319	0.908	4.690	1.777	0.629	0.484	9.639	2.311
	Private Job	4.108	0.970	4.571	1.772	0.624	0.485	9.303	2.422
	Retired	4.164	1.036	4.873	1.735	0.573	0.497	9.609	2.366
	Non-working	4.097	1.036	4.268	1.703	0.599	0.491	8.964	2.282
	Other	4.238	0.878	4.378	1.579	0.616	0.488	9.232	2.086
Chronic diseases	No	4.100	0.981 *	4.445	1.727 *	0.622	0.485	9.167	2.335
	Yes	4.217	1.000	4.913	1.783	0.630	0.484	9.761	2.367
Have children	No	4.011	0.996 *	4.404	1.727 *	0.610	0.488	9.026	2.350 *
	Yes	4.280	0.940	4.644	1.748	0.644	0.479	9.568	2.298

*P-value < 0.05

### Multivariate logistic regression of the participants’ characteristics to having adequate knowledge of vitamin D

In the analysis encompassing nine variables, only one predictor emerged as statistically significant for predicting good knowledge of Vitamin D (p-value < 0.05). Participants aged more than 60 years exhibited a substantially higher likelihood of possessing adequate knowledge about Vitamin D, approximately 8 times more than participants aged between 18 and 28 years (Table 4).

**Table 4:** Multivariate logistic regression of the participants’ characteristics to having adequate knowledge of vitamin D

Variable	Categories	*P*-value	AOR (Adjusted Odds Ratio)	95% C.I. for EXP(B)	
				Lower	Upper
Age groups	18–28	Ref			
	29–39	0.039	1.520	1.022	2.259
	40–50	0.002	2.264	1.336	3.837
	51–60	0.054	2.214	0.987	4.966
	More than 60	0.012	7.956	1.575	40.180
Gender	Female	Ref			
	Male	0.613	1.082	0.797	1.469
Living location	Countryside	Ref			
	City	0.262	0.865	0.671	1.115
Marital status	Single	Ref			
	Married	0.783	1.072	0.655	1.754
	Widowed	0.696	0.804	0.269	2.406
	Divorced	0.733	0.848	0.329	2.184
Level of education	Illiterate	Ref			
	Primary school	0.275	2.690	0.455	15.922
	Middle school	0.752	1.256	0.306	5.153
	secondary school	0.204	2.393	0.623	9.198
	University	0.210	2.344	0.618	8.888
	PhD	0.062	4.092	0.930	18.007
Monthly income	High	Ref			
	Good	0.737	0.847	0.321	2.236
	Middle	0.976	1.015	0.387	2.664
	Low	0.982	0.988	0.341	2.860
Occupation	Public sector	Ref			
	Student	0.313	1.271	0.798	2.025
	Private Job	0.510	0.865	0.563	1.331
	Retired	0.134	0.494	0.196	1.244
	Non-working	0.212	0.737	0.456	1.191
	Other	0.464	1.291	0.652	2.554
Chronic diseases	No	Ref			
	Yes	0.695	1.109	0.662	1.858
Have children	No	Ref			
	Yes	0.443	1.217	0.736	2.012

## Discussion

Over the past decade, vitamin D status has garnered significant attention in health and biomedical research, leading to a surge in studies exploring its effectiveness, applications, and deficiencies [25–27]. Notably, vitamin D deficiency has reached pandemic proportions globally, affecting both children and adults [28, 29].

Our study aligns with previous research, highlighting a widespread awareness among participants regarding the beneficial effects of vitamin D. A substantial majority (91.4% and 84.6%, respectively) recognized the essential role of vitamin D in maintaining calcium and phosphates, making it valuable in treating bone diseases and rickets.

A recent study among Syrian adults in Damascus city reported similar results to our findings, with 75.8% of participants demonstrating good knowledge about the benefits of vitamin D. They agreed that it plays a significant role in maintaining the balance of calcium and phosphate and is crucial in treating rickets and other bone diseases [30]. Consistently, a study in Saudi Arabia found that over two-thirds of the population demonstrated sufficient knowledge about the benefits of vitamin D [20].

In our study, more than two-thirds of participants demonstrated awareness of the benefits of vitamin D in maintaining healthy bones and muscles, reflecting a commendable understanding of the nutrient’s importance. Interestingly, these percentages surpassed those found in a study conducted in Australia (43.1%) but fell slightly below the figures reported in a study from France (78.1%) [31].

Contrastingly, a review in Iraq found a significant rate of vitamin D deficiency among the population, possibly due to a lack of knowledge and awareness about vitamin D sources, benefits, factors affecting levels in the body, and the consequences of deficiency on overall health [32].

Despite being a significant global health concern, a substantial number of individuals remain unaware of the crucial importance of vitamin D or how to ensure an adequate intake of this vital nutrient. A study in the United Kingdom revealed that more than two-thirds of respondents lacked a comprehensive understanding of the most common indications of vitamin D deficiency [26]. Similarly, in India, a study involving antenatal mothers found that over half of the participants had a limited understanding of vitamin D deficiency [33]. In Saudi Arabia, research discovered that only 40% of mothers visiting primary health centers in Al-Agha were aware of the benefits of providing vitamin D supplementation to their infants [34].

The Eastern Mediterranean region faces a considerable challenge concerning vitamin D deficiency, with prevalence rates reaching 58.9% in Kuwait among individuals aged 10 or older, and affecting 44.3% of those aged 18–55 in Oman. Cultural practices, such as wearing veils that limit sun exposure, contribute significantly to this high prevalence. Other potential factors include insufficient vitamin D supplementation, variations in skin pigmentation, and socioeconomic status.

Moreover, vitamin D deficiency has been associated with chronic diseases in Egyptian adults, adolescent girls in Iran and Saudi Arabia, anemia in adults residing in Riyadh, Saudi Arabia, and the influence of fast-food diets in the UAE [35, 36].

This study sheds light on significant gaps in understanding among respondents. A notable 62% disagreed with the statement that vitamin D is present only in animal meat and not in vegetables and fruits, indicating a need for improved education on this topic. Additionally, 54.1% and 54.9% of respondents believed that vegetarians are more prone to vitamin D insufficiency than non-vegetarians and that excessive sun exposure does not contribute to vitamin D poisoning, respectively.

Interestingly, these findings differ from a study in Jordan that reported good knowledge about various vitamin D sources among adults, including milk, fish, cheeses, liver, whole wheat cereals, sunlight, and mushrooms. However, this study also revealed a lack of awareness about the recommended duration of sun exposure for obtaining sufficient vitamin D [37].

These disparities emphasize the importance of addressing misconceptions and enhancing public awareness regarding vitamin D sources and their potential health implications. The study identified significant associations between higher benefits scores and specific demographics, with individuals over 60, women, and those with a Ph.D. exhibiting notably stronger connections with elevated benefits scores. This underscores the need for targeted educational initiatives to address vitamin D knowledge gaps across diverse demographic groups.

In Australia and the United Kingdom, studies reported varying demographic indicators of knowledge. The Australian study identified females, the elderly, and individuals with advanced degrees as having higher intelligence markers [38]. Conversely, the UK study found that senior individuals exhibited lower levels of knowledge, while another study by Al-Agha et al. observed no association between knowledge levels and educational attainment [26, 39].

A notable prior study focused on mothers of young children at a primary healthcare center in Cairo, revealing that over three-quarters of the mothers had a poor knowledge score regarding vitamin D and its supplementation. This may be attributed to the fact that these mothers typically sought medical advice only when issues arose with their infants, and most doctors had limited time to discuss vitamin D topics with them. Furthermore, the study identified that higher education levels and an age around 25 years were associated with the highest levels of knowledge about vitamin D among these mothers [40].

## Limitations

This research study is subject to certain limitations that warrant consideration. The utilization of an online questionnaire may introduce reporting bias, as it primarily targets community members with education, internet access via smartphones, and predominantly urban residency. Consequently, there exists a risk of selection bias, potentially limiting the generalizability of the findings. Additionally, the cross-sectional design employed in this study inherently lacks the capacity to establish causation, emphasizing the need for caution in interpreting relationships between variables. We acknowledge a potential limitation in the geographic scope of our study, as the sample was drawn exclusively from regions under the control of the Syrian Government. This restriction may impact the generalizability of our findings to areas beyond governmental control. Conducting research in conflict zones presents unique challenges, including restricted access to certain regions. We recognize the importance of considering the broader Syrian population and emphasize the need for caution when interpreting the applicability of our results to regions not covered by our study. Future studies with a more extensive geographic reach are warranted to enhance the comprehensiveness and generalizability of findings across diverse regions within Syria.

## Conclusion

The research underscores a generally sufficient understanding of vitamin D among individuals, yet identifies noteworthy gaps in knowledge, particularly in lesser-known aspects such as its role in muscle health, dietary sources, and potential consequences of excessive sun exposure. Notable correlations were found between vitamin D literacy and years of schooling, years lived, and gender. While recognizing the essential role of vitamin D in promoting healthy bones, muscles, and the immune system, the study highlights the need for enhanced public education on its dietary sources and documented health advantages. Collaborative efforts involving government health-care agencies, non-governmental organizations (NGOs), and social workers are crucial for disseminating information about the many benefits of vitamin D to children and their families. Moreover, the study suggests the necessity for interventions and qualitative investigations to assess the current state of knowledge, elucidate the reasons for the observed low awareness levels, and address the identified knowledge gaps. These collective efforts aim to empower the Syrian population with improved vitamin D knowledge, consequently mitigating health issues arising from deficiencies or excesses of this crucial hormone.

### Electronic supplementary material

Below is the link to the electronic supplementary material.

Supplementary Material 1

## Data Availability

The datasets used and/or analysed during the current study available from the corresponding author on reasonable request.

## Declarations

### Data collection group

1- Tony Aldawoud: Damascus University, Faculty of medicine, Damascus, Syria, tonydawoud7@gmail.com.

2- Nour Ali Al-Fahed: Tishreen University, Faculty of medicine, nouralfahed22@gmail.com.

3- lama ammar ibrahem: Damascus University, Faculty of medicine, Damascus, Syria, laimouna455@gmail.com.

4- Doaa Bayoun: Damascus University, Faculty of medicine, Damascus, Syria, doaabayoun@gmail.com.

5- Doaa Salamah: Tishreen University, Faculty of medicine, do3a2sal2000@gmail.com.

6- Lina AlBougha: Damascus University, Faculty of medicine, Linaalbougha998@hotmail.com.

8- Farah Altaher: Aleppo University, Faculty of medicine, farahtaher2000@gmail.com.

9- Nermen Bouti: Damascus university faculty of medicine, nermen.buti@gmail.com.
