# Supplementary material for: Assessment the awareness of vitamin D deficiency among the general population in Syria: an online cross-sectional study
Source: BMC Public Health. 2024 Apr 1;24:938. doi: 10.1186/s12889-024-18376-2 (PMC10985847; doi:10.1186/s12889-024-18376-2)
Supplement: Supplementary file 1 — Supplementary Material 1 [file 12889_2024_18376_MOESM1_ESM.docx]

Supplementary Table 1: Socioeconomic demographic information of the participants that completed the survey.

| Variable | Categories | N | % |
| --- | --- | --- | --- |
| Age | Mean ± SD | 30.80 ± 11.957 | |
| Age groups | 18–28 | 1521 | 57.9% |
|  | 29–39 | 517 | 19.7% |
|  | 40–50 | 380 | 14.5% |
|  | 51–60 | 144 | 5.5% |
|  | More than 60 | 63 | 2.4% |
| Gender | Female | 1955 | 74.5% |
|  | Male | 670 | 25.5% |
| Living location | Countryside | 1096 | 41.8% |
|  | City | 1529 | 58.2% |
| Marital status | Single | 1443 | 55.0% |
|  | Married | 1071 | 40.8% |
|  | Widowed | 53 | 2.0% |
|  | Divorced | 58 | 2.2% |
| Level of education | Illiterate | 14 | 0.5% |
|  | Primary school | 43 | 1.6% |
|  | Middle school | 117 | 4.5% |
|  | secondary school | 583 | 22.2% |
|  | University | 1707 | 65.0% |
|  | PhD | 161 | 6.1% |
| Monthly income | High | 47 | 1.8% |
|  | Good | 879 | 33.5% |
|  | Middle | 1514 | 57.7% |
|  | Low | 185 | 7.0% |
| Occupation | Student | 843 | 32.1% |
|  | Public sector | 429 | 16.3% |
|  | Private Job | 716 | 27.3% |
|  | Retired | 110 | 4.2% |
|  | Non-working | 362 | 13.8% |
|  | Other | 164 | 6.3% |
| Chronic diseases | No | 2349 | 89.5% |
|  | Yes | 276 | 10.5% |
| Have children | No | 1637 | 62.4% |
|  | Yes | 988 | 37.6% |

Supplementary Table 2: Participants' awareness toward knowledge of advantages and source of vitamin D and outcomes of vitamin D toxicity.

| Variable | Categories | N | % |
| --- | --- | --- | --- |
| Vitamin D is used to treat bone disease and rickets | No | 225 | 8.6% |
|  | Yes | 2400 | 91.4% |
| Vitamin D is important in the maintenance of calcium and phosphates | No | 405 | 15.4% |
|  | Yes | 2220 | 84.6% |
| Vitamin D is important in the maintenance of bone and teeth | No | 311 | 11.8% |
|  | Yes | 2314 | 88.2% |
| Vitamin D helps to strengthen immunity | No | 528 | 20.1% |
|  | Yes | 2097 | 79.9% |
| Vitamin D helps to strengthen muscles | No | 861 | 32.8% |
|  | Yes | 1764 | 67.2% |
| Sun exposure encourages vitamin D production in the skin | No | 115 | 4.4% |
|  | Yes | 2510 | 95.6% |
| Vitamin D is found in animal meat but not in vegetables and fruits | No | 1633 | 62.2% |
|  | Yes | 992 | 37.8% |
| People residing in cloudy areas are more prone to vitamin D deficiency | No | 358 | 13.6% |
|  | Yes | 2267 | 86.4% |
| Frequent sun exposure does not lead to vitamin D poisoning | No | 1205 | 45.9% |
|  | Yes | 1420 | 54.1% |
| Use of sunscreen creams may be a cause of vitamin D deficiency | No | 1614 | 61.5% |
|  | Yes | 1011 | 38.5% |
| A fat-free diet may be a cause of vitamin D deficiency | No | 1240 | 47.2% |
|  | Yes | 1385 | 52.8% |
| Dark skin is more prone to vitamin D deficiency than fairer skin | No | 1854 | 70.6% |
|  | Yes | 771 | 29.4% |
| Vegetarians are more likely to have vitamin D deficiency than nonvegetarians | No | 1183 | 45.1% |
|  | Yes | 1442 | 54.9% |
| Consequences of vitamin D toxicity in Hypercalcemia | No | 990 | 37.7% |
|  | Yes | 1635 | 62.3% |
